# Recent advances in stroke biomarkers – implications for prognosis and treatment

**DOI:** 10.1097/WCO.0000000000001450

**Published:** 2025-12-02

**Authors:** Jessica Seetge, Johannes Frenger, Mira Katan, Gerrit M. Grosse

**Affiliations:** a Department of Neurology and Stroke Center, University Hospital Basel; b Department of Clinical Research, University of Basel, Basel, Switzerland

**Keywords:** acute ischemic stroke, blood biomarkers, diagnosis, etiology, prognosis

## Abstract

**Purpose of review:**

To summarize recent advances in blood-based biomarkers for acute ischemic stroke relevant to diagnosis, etiological assessment, risk prediction, and outcome prognostication, and to outline future directions for clinical implementation.

**Recent findings:**

Novel biomarkers enhance differentiation of ischemic from hemorrhagic stroke and large vessel occlusion detection, optimizing triage via point-of-care testing. Specific biomarkers improve etiological classification and identification of mechanisms like cardioembolic sources and atrial cardiopathy, enabling targeted secondary prevention. Circulating markers stratify risks of vascular recurrence and infections, linking inflammatory, thrombotic, and endothelial pathways. Prognostic biomarkers refine predictions of functional outcomes, mortality, and reperfusion responses.

**Summary:**

To translate these promising findings into clinical care and to identify novel molecular targets, standardized sample collection, rigorous external validation, and multiomics/panel integration will be required. In this sense, blood-based-biomarkers have the potential to sustainably improve diagnostics, prognosis and treatment in stroke care.

## INTRODUCTION

**FIGURE 1:**
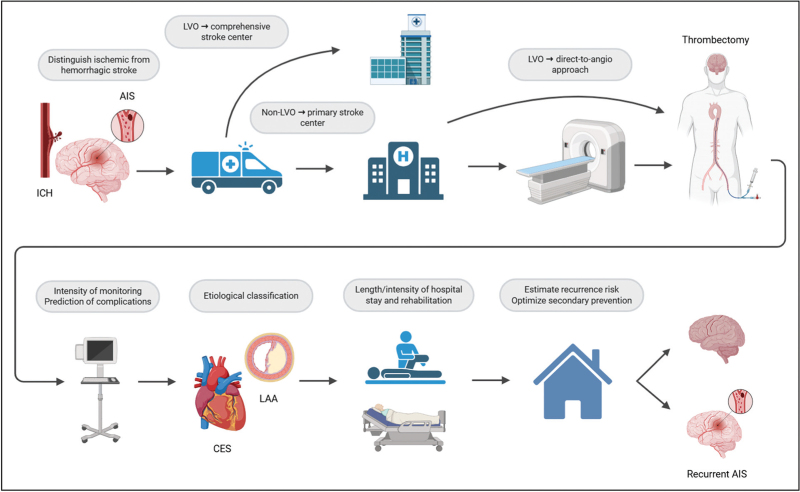
How blood based biomarkers could improve prehospital, hospital and long-term workup in acute ischemic stroke (created in BioRender. Frenger J. (2025) https://BioRender.com/1s825vy). AIS, acute ischemic stroke; CES, cardioembolic stroke; ICH, intracranial hemorrhage; LAA, large artery atherosclerosis.

Despite the extensive investigation of numerous blood-based biomarkers in acute ischemic stroke (AIS), none have yet been successfully implemented into routine clinical practice. In this review, we provide a critical and forward-looking overview of the most recent advances in biomarker research, highlighting both key limitations as well as promising potential for translation into clinical application (Fig. 1). 

**Box 1:**
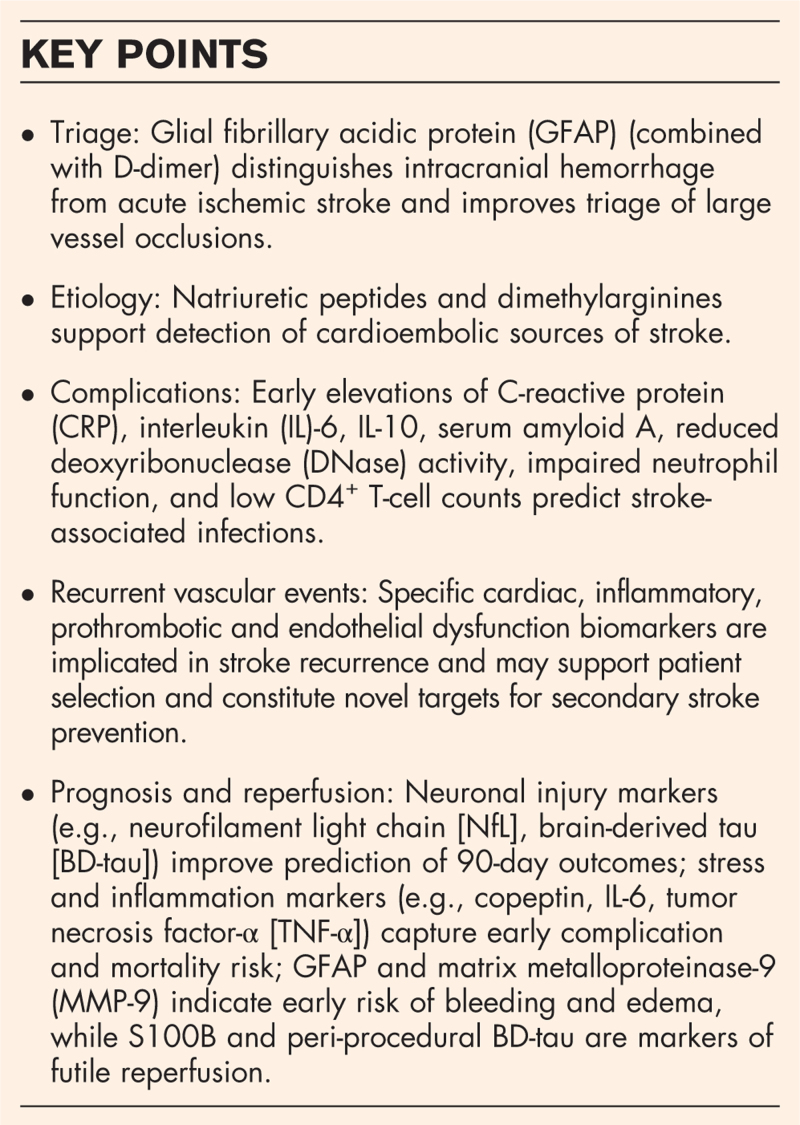
no caption available

### Biomarkers for supporting diagnostic workup of stroke

Rapid and accurate diagnosis is essential to ensure optimal treatment in acute stroke. First, AIS must be distinguished from intracranial hemorrhage (ICH) as the according treatment strategies differ fundamentally. The time is brain paradigm also affects ICH as highlighted by the latest trials wherein patients receiving early hematoma evacuation or early blood pressure lowering had better functional outcome [1^▪^,2^▪^]. These findings underscore the need to implement acute, rapid-care bundles for ICH [3^▪^]. Second, large vessel occlusion (LVO) should be identified and differentiated from non-LVO cases to enable timely transfer of the patient to the most appropriate facility, i.e. a comprehensive stroke center with thrombectomy capability. Point-of-care testing (POCT) devices hold great potential especially in the prehospital setting, as they could significantly reduce the time to recanalization, which in turn has a major impact on functional outcomes after ischemic stroke.

In the context of differentiating AIS from ICH, several biomarkers reflecting neurovascular inflammation, endothelial integrity, neuronal injury, and cardiac dysfunction have been investigated. Among these, glial fibrillary acidic protein (GFAP) – an intermediate filament protein primarily expressed in astrocytes – has emerged as one of the most promising candidates. Following stroke, GFAP is released into the bloodstream as a consequence of blood–brain barrier (BBB) disruption [4]. Patients with ICH typically exhibit higher GFAP levels, likely due to earlier and more extensive BBB breakdown. A recently published systematic review and meta-analysis reported a sensitivity and specificity of GFAP measured 3 h after symptom onset of 73% [37–94%] and 97% [82–100%], respectively [5]. Biomarker panels have also been explored: in a study of 189 patients with AIS and ICH enrolled within 4.5 h after onset, a panel including retinol binding protein (RBP-4), N-terminal pro-B-type natriuretic peptide (NT-proBNP), and GFAP achieved a specificity of 100% for AIS detection. When stroke mimics were included, specificity decreased slightly to 96.8% [6].

In the context of LVO detection, a large prospective observational cohort study identified early D-dimer levels as independent predictors of LVO, the addition of high D-dimer levels to the clinical National Institutes of Health Stroke Scale (NIHSS) >10 led especially to a high specificity of 93% and therefore fourfold reduction of false positives [7]. Another prospective observational cohort study investigating the combination of D-dimer and GFAP as the optimal measure for LVO diagnosis. The incorporation of GFAP into algorithms with D-dimer and clinical scales contributed to maintaining high specificity, particularly by effectively excluding patients with hemorrhagic stroke, in whom GFAP levels are markedly elevated. In 382 patients, the combined measurement of GFAP and D-dimer achieved a high precision for LVO, with sensitivity of 71% and specificity of 91% and therefore validated the results of a previously published retrospective study [8^▪▪^,^9^]. Recently, the diagnostic thresholds for both biomarkers have been further validated using POCT, enabling earlier and more rapid assessment in preclinical settings [10^▪^]. Ongoing research is evaluating whether POCT may also facilitate direct-to-angiography suite triage by circumventing standard imaging acquisition for appropriately selected patients.

Several challenges complicate the comparison of existing studies, including differences in reference groups (healthy controls versus stroke mimics), variability in sampling time points, small sample sizes, and the lack of standardized reporting. Further validation studies are needed before clinical implementation.

### Biomarkers for identification of underlying stroke etiology

Identifying the underlying etiology of stroke is crucial for optimal secondary prevention. Yet, challenges remain: up to one-third of AIS patients are classified as having undetermined etiology by the Trial of Org 10172 in Acute Stroke Treatment (TOAST) criteria or as embolic stroke of unknown source (ESUS) [11^▪▪^,^12^]. Trials have not shown general superiority for oral anticoagulation (OAC) over aspirin in ESUS, highlighting the need for a more accurate attribution [13,14]. Treating patients when the cause is unknown or multiple causes exist remains a critical issue. Blood biomarkers offer promise to uncover underlying stroke mechanisms and associated risk factors, with significant advances already made using natriuretic biomarkers to detect cardioembolic stroke (CES) causes, but also for the detection of other subtypes such as large artery atherosclerosis.

B-type natriuretic peptide (BNP) is primarily synthesized in the ventricular myocardium, while atrial natriuretic peptide (ANP) is produced in the atrium. Both hormones circulate as prohormones (e.g. NT-proBNP) and active forms (ANP, BNP) and are secreted in response to myocardial stretch. ANP levels reflect left atrial pressure, whereas BNP levels are linked to left ventricular pressure [15]. A meta-analysis compared the prognostic value of BNP and NT-proBNP for detecting covert atrial fibrillation (AF) after AIS. NT-proBNP showed superior diagnostic accuracy, likely due to its longer half-life, which allows better detection of paroxysmal AF [16]. The change in BNP levels represents another interesting marker, as a substantial proportion of patients present with paroxysmal AF, and conversion to sinus rhythm may reduce BNP concentrations. In a retrospective cohort study, ΔLog BNP demonstrated a stronger association with AF than established markers such as absolute BNP levels. These findings require external validation [17^▪^]. Another meanwhile established biomarker is ANP, secreted by the left atrium and potentially more specific for atrial cardiopathy and AF. Its precursor, mid-regional pro-atrial natriuretic peptide (MR-proANP), has a longer half-life, making it easier and more reliable to measure [18]. In a prospective study with external validation, MR-proANP proved useful for identifying patients at risk of newly diagnosed AF and guiding decisions on prolonged monitoring as well as for estimating of recurrence risk [19]. The results of a recently published systematic review and meta-analysis underline the predictive abilities showing moderate to high discriminative ability for the identification of CES and significantly improving the diagnostic value of clinical models [20^▪^].

Natriuretic peptides may not only aid in diagnosing CES but also help guide OAC in patients with high cardioembolic risk and signs of atrial cardiopathy. The ARCADIA trial, comparing apixaban to aspirin in ESUS patients with atrial cardiopathy (NT-proBNP >250 pg/ml), showed no benefit of apixaban over aspirin [21^▪▪^]. Possible reasons include delayed randomization (up to two months poststroke) and a low NT-proBNP cutoff, enrolling patients without significant atrial cardiopathy. Thus, ARCADIA may not clarify whether markedly elevated natriuretic peptides justify anticoagulation. The currently ongoing MOSES trial (NCT03961334) uses MR-proANP, randomizes patients within 7 days, and selects them solely by biomarker levels in a pragmatic approach. Of note, cardiac biomarkers also have a connection to dementia [22].

Not only cardiac biomarkers are associated with CES. Endothelial biomarkers such as symmetric dimethylarginine (SDMA) correlate independently with AF detection rates and with left atrial volume index (a marker of atrial cardiopathy), therefore serving as additional markers that could guide the duration of cardiac monitoring [23,24^▪^] or even anticoagulation.

However, the cause of a first ischemic stroke may differ from that of subsequent events. Recurrent strokes can arise from other mechanisms, highlighting the need to consider competing risks.

### Biomarkers for stroke recurrences and complications

Patients with AIS are at high risk for recurrent vascular events as well as other severe complications impacting overall prognosis [25]. Biomarkers have the potential to contribute significantly to the stratification of these risks, thereby identifying patients who would benefit from either intensified monitoring or even early preventive measures.

#### Biomarkers of stroke recurrence

Specific circulating biomarkers may be useful not only for risk stratification of recurrent vascular events but also as actual therapeutic targets. This concerns particularly inflammatory mediators central to stroke pathophysiology. Mechanisms of inflammation and thrombosis interact through immunothrombosis, e.g. by neutrophil extracellular traps (NETs) constituting a link between immune activation and clot formation [26^▪^]. NETs, which are composed of cell-free DNA (cfDNA), histones, and proteins, promote thrombosis and are frequent in cerebral thrombi [27]. Circulating NET markers such as cfDNA and myeloperoxidase-histone complexes correlate with thrombus NET content, indicating potential in assessing thrombotic mechanisms [28]. Furthermore, recent evidence from translational work shows that cfDNA released during stroke activates the AIM2 inflammasome in atherosclerotic plaques, with subsequent destabilization and increased recurrence risk. Intriguingly, targeting cfDNA using deoxyribonuclease (DNase) proved beneficial for lowering risks of recurrences in an experimental setting [29^▪▪^].

The interleukin (IL)-1β–IL-6–C-reactive protein (CRP) axis is central in stroke pathophysiology and turned out to be associated with recurrent strokes, not only restricted to atherothrombotic events [30^▪^,^31^]. Anti-inflammatory agents such as canakinumab and colchicine demonstrated potential in vascular event prevention by targeting the inflammatory pathway involving IL-1β and the NLRP3 inflammasome, respectively [32,33,34^▪▪^,^35^]. Monitoring IL-1β–IL-6–CRP could optimize patient selection and treatment monitoring, as recently shown in a subanalysis of the CONVINCE trial [36].

Ongoing clinical trials currently explore secondary prevention via IL-6 inhibition (ziltivekimab) and DNase therapy (Dornase Alfa). Moreover, large-scale collaborative biomarker cohorts will contribute to the identification of entirely new molecular targets in the coming years.

#### Biomarkers for prediction of stroke-associated infections

Stroke-associated infections (SAI) are substantially worsening functional outcomes following stroke and are increasing risk for death [37]. A general application of antibiotics in stroke patients did not prove successful and therefore improved risk stratification is highly warranted.

SAI develop as a result of immunosuppression, which is triggered by excessive activation of the sympathetic nervous system and the hypothalamic-pituitary-adrenal axis and is characterized by rapid lymphopenia, impaired T-cell function, a shift toward TH2 responses, and dysfunction of innate immune mechanisms [37]. These mechanisms consequently compromise antibacterial defense and predispose to infections, particularly stroke-associated pneumonia (SAP) and urinary tract infections (UTI) [37].

Among circulating molecular biomarkers for predicting SAI, CRP and IL-6 are the most extensively investigated [38]. Early poststroke CRP and IL-6 levels have been independently linked to SAI [39] and specifically to outcome-relevant lower respiratory tract infections [40]. Likewise, the anti-inflammatory cytokine IL-10 proved to be predictive of subsequent systemic infections, independent of stroke severity and extent of brain injury [39,41].

Copeptin has been identified as a robust biomarker for prediction various poststroke complications including SAI, which may support decision-making in clinical practice [42]. More recently, serum amyloid A has emerged as another independent predictor of SAI [43].

In the hyperacute phase, reduced endogenous DNase activity which is degrading cfDNA has been found in thrombectomy patients who later developed SAI compared to those who did not [44]. This finding is of particular interest since DNase is available as a recombinant drug and is currently being tested as therapeutic to improve reperfusion and secondary prevention in stroke.

Impaired neutrophil function, particularly diminished phagocytosis and oxidative bursts, has been shown to predict the occurrence of SAI independently of clinical risk factors and stroke severity [45]. Similarly, lower CD4^+^ T cell counts measured the day after stroke may serve as an early indicator of SAI [46].

Although no single biomarker currently achieves adequate predictive accuracy for routine use, integrating biomarker panels with established clinical risk factors may improve early identification of high-risk patients, support targeted prevention, reduce unnecessary antibiotic use, and enhance outcomes. Prospective validation and standardization of such biomarker-guided strategies remain essential before they can be widely implemented in clinical practice.

#### Biomarkers of cardiac injury

Patients with AIS face a high risk for myocardial injury. Cardiac troponins (cTnI, cTnT) are the principal biomarkers for detecting cardiac injury and myocardial infarction in acute stroke [47]. Interestingly, temporal dynamics of troponin concentrations following stroke do not imply myocardial infarction and therefore do not reflect the type of myocardial injury [48^▪▪^].

### Biomarkers for prognostication of stroke outcomes

Prognosis after AIS is mainly determined by age, baseline severity, lesion topography, and early complications. Specific blood-based biomarkers can refine these estimates in the early course when interpreted with attention to sampling timing, reperfusion status, preexisting comorbidities, and stroke subtype.

#### Cardio-cerebral stress markers

Among routine markers, natriuretic peptides most consistently relate to mortality [49^▪^]. As outlined above, these biomarkers are frequently elevated in CES, a subtype associated with higher stroke severity and poorer long-term survival [50^▪^]. Consequently, apparent associations with 90-day functional independence weaken once etiology is accounted for. In practice, these peptides are useful for long-term risk stratification and secondary prevention but add little to short-term decisions driven by lesion location and early complications.

Copeptin, a stable surrogate of vasopressin release, captures the early neurohumoral stress response [51]. When measured within 24–72 h, higher concentrations are associated with worse functional outcome, early mortality, and a composite of complications [pneumonia, malignant edema, seizures] [52^▪^]. These associations generally persist after adjustment for age and stroke severity, and adding copeptin to prognostic models improves performance beyond clinical variables alone [53^▪▪^]. Of currently studied biomarkers, copeptin stands out as one of the most promising for meaningfully refining bedside risk assessment in the hyperacute phase, supporting decisions around prolonged monitoring and early dysphagia assessment.

#### Inflammatory biomarkers

Within inflammatory signaling, IL-6 and tumor necrosis factor-α (TNF-α) consistently associate with adverse outcomes [54^▪^]. Chemokines such as CXCL10 and MCP-1 have also been linked to poorer outcomes, though findings are heterogeneous, and assays are not standardized for routine prognostication [55^▪^,56^▪^]. Levels of Fractalkine (CX3CL1), within this class, show an inverse association with severity and outcomes after AIS, potentially reflecting anti-inflammatory and cerebroprotective properties [57]. By contrast, mannose-binding lectin and procalcitonin are difficult to interpret unless infection is explicitly excluded, as apparent effects often reflect early sepsis biology.

#### Brain-damage and synaptic biomarkers

Neurofilament light chain (NfL) is released with axonal injury [58]. Concentrations are higher in AIS than in transient ischemic attack (TIA) or controls and correlate with clinical severity and infarct burden. For outcome prediction, days 3–7 sampling is more informative than admission: higher values in this window are associated with poorer 90-day functional outcomes and increased mortality [59^▪^]. Serial testing can also identify patients at risk of hemorrhagic transformation or progressive white-matter injury and inform monitoring intensity and follow-up care.

Brain-derived tau (BD-tau), a central nervous system-specific tau isoform enriched in neurons, is an emerging biomarker in the field that rises rapidly over the first 24 h after AIS, mirroring early infarct progression. At 24 h, BD-tau levels can stratify 90-day functional outcomes, performing comparably to established markers in several cohorts [60^▪^,61^▪^].

Beta (β)-synuclein is a circulating marker of synaptic damage with promising associations to functional outcomes and appears less influenced by systemic comorbidity than many conventional markers [62^▪^]. Early rises may complement axonal (NfL) and neuronal (BD-tau) levels, offering a more granular picture of parenchymal injury.

#### Biomarkers of cerebral reperfusion

Biomarkers measured before or following reperfusion therapy can help inform about the success of recanalization, hemorrhagic risk, and mechanisms that may limit treatment effects and offer therapeutic targets.

S100B peaks at 48–72 h and mirrors infarct demarcation on follow-up imaging. In thrombectomy cohorts, it remains low when large perfusion deficits are salvaged, whereas elevations despite successful angiography may indicate futile recanalization or periprocedural injury [63^▪^]. In accordance, S100B was shown to be independently associated with symptomatic brain edema and intracranial hemorrhage following AIS [64]. Periprocedural rises in BD-tau from admission to 24 h likewise suggest futile reperfusion and may complement the S100B-based prediction [65^▪▪^].

Measured within the first 24 h, GFAP may support early postreperfusion safety assessment: higher values raise concern for hemorrhagic complications, while low values, considered alongside clinical and imaging findings, can support mobilization and antithrombotic decisions [66^▪▪^]. Matrix metalloproteinase-9 (MMP-9), a well-established mediator of BBB disruption, further refines bleeding and edema risk at baseline and after reperfusion when sampled early and evaluated in clinical context [67^▪^].

#### Thrombo-inflammation and endothelial dysfunction

Low ADAMTS-13 activity and high von Willebrand factor (vWF) indicate endothelial dysregulation and a pro-thrombotic milieu, and have been associated with worse outcomes in in patients undergoing intravenous thrombolysis or mechanical thrombectomy [68,69,70^▪^], implicating the vWF–ADAMTS-13–platelet axis as a potential therapeutic target. Elevated cfDNA is independently associated with poorer outcomes and higher mortality after thrombectomy [44]. As described above, cfDNA is not only a prognostic biomarker in this regard but also a therapeutic target that can be tackled using recombinant DNase [44]. High-mobility group box-1 (HMGB1), another canonical damage-associated molecular pattern, bridges early sterile inflammation and BBB injury (relevant to thrombolysis-related hemorrhage) with later neurovascular remodeling and atherogenesis, positioning it as both risk marker and candidate therapeutic target in postreperfusion care [71^▪^].

## CONCLUSION

To realize the translational potential of these and novel biomarkers, future research should focus on harmonized multicenter studies, rigorous validation, data integration across omics and multidimensional platforms, and targeted assessment of how biomarkers may guide personalized therapeutic strategies (see Table 1). Ultimately, blood-based biomarkers are poised to transform the diagnosis and treatment of stroke by enabling earlier, more precise and tailored approaches.

**Table 1:** Overview of blood-based biomarkers and clinical utility in acute ischemic stroke

Category	Biomarker	Potential clinical application
(I) Stroke triage	GFAP	Distinguish AIS from ICH, LVO detection
	RBP-4	Distinguish AIS from ICH
	NT-proBNP	Distinguish AIS from ICH
	D-Dimer	LVO detection
(II) Stroke etiology	BNP // NT-proBNP	Identify cardioembolic stroke etiology
	ΔLog BNP	Identify cardioembolic stroke etiology
	MR-proANP	Identify cardioembolic stroke etiology
	SDMA	Identify cardioembolic stroke etiology
(III) Recurrence	MR-proANP	Recurrent vascular events
	NT-proBNP	Recurrent vascular events
	IL-6/CRP	Recurrent vascular events
	cfDNA	Recurrent vascular events
	NETs markers	Recurrent vascular events
(IV) Complications	IL-6/CRP/IL-10	Predict stroke-associated infections
	DNase	Predict stroke-associated infections
	SAA	Predict stroke-associated infections
	Troponins	Diagnose myocardial infarction in AIS
	Copeptin	Predict stroke-associated infections and seizures
(V) Outcome and prognostication	BNP // NT-proBNP	Functional outcome, mortality
	MR-proANP	Functional outcome, mortality
	Copeptin	Functional outcome, mortality, in-hospital complications
	IL-6	Functional outcome
	TNF-α	Functional outcome
	CXCL10 and MCP-1	Functional outcome
	Fractalkine	Severity, functional outcome
	NfL	Severity, functional outcome, mortality
	BD-tau	Functional outcome, futile reperfusion
	β-synuclein	Functional outcome
	S100B	Futile reperfusion
	GFAP	Hemorrhagic transformation
	MMP-9	Hemorrhagic transformation, edema
	vWFADAMTS-13	Functional outcome, reperfusion
	cfDNA	Functional outcome, mortality
	HMGB1	Hemorrhagic transformation, edema

BD-tau, brain-derived tau; BNP, B-type natriuretic peptide; cfDNA, cell-free DNA; CRP, C-reactive protein; DNase, deoxyribonuclease; GFAP, glial fibrillary acidic protein; HMGB1, high-mobility group box-1; IL, interleukin; MMP-9, matrix metalloproteinase-9; MR-proANP, mid-regional pro-atrial natriuretic peptide; NET, neutrophil extracellular trap; NfL, neurofilament light chain; NT-proBNP, N-terminal pro-B-type natriuretic peptide; RBP-4, retinol-binding protein-4; SAA, serum amyloid A; SDMA, symmetric dimethylarginine; TNF-α, tumor necrosis factor-α; vWF, von Willebrand factor.

## Acknowledgements

*None.*

### Financial support and sponsorship

*No external resources were used to prepare this work.*

### Conflicts of interest

*J.S. and J.F. report no conflicts of interest. M.K. reports nonfinancial support from Roche diagnostics and BRAHMS Thermofisher Scientific and is involved in the Advisory Boards of AstraZeneca, Bayer, BMS/Pfizer/Jansen and Medtronic. G.M.G. reports research grants from the German Ministry of Education and Research (B.M.B.F.), the European Commission and the Lower Saxony Ministry of Science and Culture (M.W.K.) as well as honoraria for advisory boards by Boehringer Ingelheim and Bayer.*

## REFERENCES AND RECOMMENDED READING

Papers of particular interest, published within the annual period of review, have been highlighted as:

▪ of special interest
▪▪ of outstanding interest
